# Re-tear after arthroscopic rotator cuff repair can be predicted using deep learning algorithm

**DOI:** 10.3389/frai.2024.1331853

**Published:** 2024-02-29

**Authors:** Zhewei Zhang, Chunhai Ke, Zhibin Zhang, Yujiong Chen, Hangbin Weng, Jieyang Dong, Mingming Hao, Botao Liu, Minzhe Zheng, Jin Li, Shaohua Ding, Yihong Dong, Zhaoxiang Peng

**Affiliations:** ^1^Ningbo University affiliated Li Huili Hospital, Ningbo University, Ningbo, China; ^2^Health Science Center, Ningbo University, Ningbo, China; ^3^Faculty of Electrical Engineering and Computer Science, Ningbo University, Ningbo, China; ^4^Key Laboratory of Mobile Network Application Technology of Zhejiang Province, Ningbo University, Ningbo, China

**Keywords:** deep learning, rotator cuff retear, graph convolution network, prediction model, big data

## Abstract

The application of artificial intelligence technology in the medical field has become increasingly prevalent, yet there remains significant room for exploration in its deep implementation. Within the field of orthopedics, which integrates closely with AI due to its extensive data requirements, rotator cuff injuries are a commonly encountered condition in joint motion. One of the most severe complications following rotator cuff repair surgery is the recurrence of tears, which has a significant impact on both patients and healthcare professionals. To address this issue, we utilized the innovative EV-GCN algorithm to train a predictive model. We collected medical records of 1,631 patients who underwent rotator cuff repair surgery at a single center over a span of 5 years. In the end, our model successfully predicted postoperative re-tear before the surgery using 62 preoperative variables with an accuracy of 96.93%, and achieved an accuracy of 79.55% on an independent external dataset of 518 cases from other centers. This model outperforms human doctors in predicting outcomes with high accuracy. Through this methodology and research, our aim is to utilize preoperative prediction models to assist in making informed medical decisions during and after surgery, leading to improved treatment effectiveness. This research method and strategy can be applied to other medical fields, and the research findings can assist in making healthcare decisions.

## 1 Introduction

Rotator cuff tears (RCTs) are a common cause of shoulder pain and often leads to dysfunction in the glenohumeral joint (Rachelle and Romi, 2021). They affect over 40% of patients over the age of 60, resulting in annual surgical repairs ranging from 30,000 to 75,000 in the USA (Ricchetti et al., 2012). In addition to pain and dysfunction, Rotator cuff injuries can have negative effects on mental and social wellbeing (Chepeha and Sheps, 2016). The high prevalence of this disease results in significant socioeconomic burden and strains on healthcare insurance due to the associated costs of diagnosis, treatment, and rehabilitation (Yamamoto et al., 2010). Arthroscopic rotator cuff repair (ARCR) of shoulder is an effective and the most common operative treatment. It alleviates the pain and functional impairments caused by this disease after surgery and rehabilitation exercises. However, postoperative complications can significantly impact the effectiveness of the treatment for this condition. Rotator cuff retear is a prominent post-arthroscopic rotator cuff repair (ARCR) complication, with a significant risk of retear ranging from 8.3% to 27.3% (Fu et al., 2020; Davey et al., 2023; Routledge et al., 2023; Tsuchiya et al., 2023). Tear recurrence can be influenced by factors including: (1) inadequate strength of the initial repair construct, and (2) inappropriate postoperative rehabilitation causing structural failure of the repair (Bigliani et al., 1992; Neviaser and Neviaser, 1992; Boileau et al., 2005; Cho et al., 2015). Once retear occurs, it results in persistent pain and limited mobility in the shoulder joint, even more severe than before surgery. Doctors have only two treatment options: conservative management and revision surgery. However, medication (i.e., conservative management) has limited efficacy. A 10-year follow-up study on patients with postoperative re-tear of ARCR managed conservatively found no improvement in the average long-term ASES score (79 points, range 50–95 points) or the average visual analog scale (VAS) pain score (2.2 points, range 1–4 points) compared to pre-treatment scores (Paxton et al., 2013). Additionally, revision surgery is challenging and has a high retear rate (Desmoineaux, 2019). Reverse shoulder arthroplasty can indeed address the problem, but it entails substantial trauma and a multitude of complications (Ernstbrunner et al., 2017). A retrospective study by Jeong et al. (2022), involving 200 patients, revealed a gradual decline in long-term functional outcomes of the shoulder joint over time. In cases of postoperative retear, conservative medication treatment or even surgical revision fail to yield satisfactory outcomes. Meanwhile, the patient's symptoms continue to worsen, significantly impairing the effectiveness of medical care and giving rise to potential doctor-patient conflicts. Consequently, our focus has shifted to the prevention of postoperative re-tear.

In the past decades, artificial intelligence (AI) has made significant progress and demonstrated immense potential in addressing a wide range of problems. The medicine field is currently exploring the clinical applications of AI. AI is one of the new-age computer technologies that can perform human cognitive functions by analyzing data. Deep learning is an application of artificial intelligence, utilizes big data analysis to generate predictive algorithms (Obermeyer and Emanuel, 2016). When dealing with complex data problems, and even certain socially relevant data problems (such as medical issues), it demonstrates excellent performance (Jordan and Mitchell, 2015). Suzuki et al. (2022) achieved 99.3% accuracy, comparable to orthopedic surgeons, in diagnosing radius fracture from plain radiographs using a CNN model. Mutasa et al. (2020), Bae et al. (2021) and Urakawa et al. (2019) in their respective studies used DL models to diagnose femoral neck fracture and intertrochanteric fracture, both of which were superior to specialist orthopedic surgeons. Deep learning can also predict osteoporosis and assess fracture risk (Hsieh et al., 2021; Löffler et al., 2021). Furthermore, researchers have developed models for image recognition of rotator cuff injuries and calcific tendinitis (Chiu et al., 2022; Lee et al., 2023). Recent studies have even established prediction models for femoral head necrosis, thereby aiding in the formulation of clinical decisions (Shen et al., 2024). This expands the capabilities of doctors in their practice. The prospect of artificial intelligence medical models is worthy of recognition, and its application scope is constantly expanding (Feng et al., 2022).

The study aims to utilize deep learning algorithms to identify numerous preoperative variables and construct a postoperative re-tear prediction model for ARCR. The goal is to predict the likehood of re-tear before surgery, enabling clinicians to make informed decisions such as selecting a more secure fixation method during the operation or implementing a more stable rehabilitation approach post-operation, thereby significantly reducing the probability of re-tear. This approach can improve the treatment effectiveness of ARCR, enhance the doctor-patient relationship, and optimize the diagnosis and treatment system. There has been no attempt made to predict the rate of re-tear after ARCR. First, we performed this experiment under the assumption that postoperative retear could be predicted using various preoperative indicators.

## 2 Materials and methods

### 2.1 Study subjects and data collection

**Figure 1:**
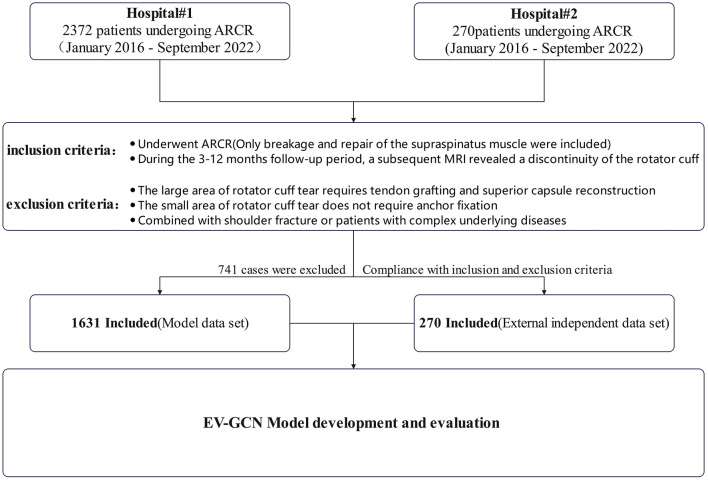
Flow chart of inclusion and exclusion.

Subjects were patients who underwent ARCR by four experienced chief surgeons in our hospital from January 2016 to September 2022. The inclusion criteria are defined as follows: patients who underwent ARCR (only breakage and repair of the supraspinatus muscle were included); during the 3–12 months follow-up period, a subsequent MRI revealed a discontinuity of the rotator cuff. The exclusion criteria are defined as follows: the large area of rotator cuff tear requires tendon grafting and superior capsule reconstruction; the small area of rotator cuff tear does not require anchor fixation; combined with shoulder fracture or patients with complex underlying diseases such as head injury caused by a car accident and heart, liver, lung and other multiple organ dysfunction etc. (Figure 1).

All data were collected from the electronic medical record and clinical data records of the Affiliated Lihuili Hospital, Ningbo University. External data were obtained from East Medical Center. In this study, research group is defined as patients with rotator cuff discontinuity occurring within 3–12 months after surgery (identified through rotator cuff tissue discontinuity observed during MRI re-examination). The control group was defined as no difference at 1-year follow-up. A total of 62 preoperative variables were collected (Table 1).

**Table 1:** A total 62 types of preoperative descriptive data.

**Types of variables**	**Variables**	**Range**	**Units**
Clinical index	Side affected	Right/Left	NA
	Caused by trauma	Yes/No	NA
	Symptom duration	7 d;7–30 d;1–3m;3–6 m;6–12 m;1a	NA
	Nocturnal pain	With/Without	NA
Demographics	Sex	Male/Female	NA
	Age	16–85	Year
Comorbidities	Hypertension	With/Without	NA
	Hyperlipemia	With/Without	NA
	Diabetes mellitus	With/Without	NA
	Smoking history	With/Without	NA
	Drinking history	With/Without	NA
Laboratory index	RBC	3.80–5.10	^*^10^∧^12/L
	HB	115–150	g/L
	HCT	35.0–45.0	%
	PLT	125–350	^*^10^∧^9/L
	PCT	0.11–0.28	%
	TP	65.0–85.0	g/L
	ALB	40.0–55.0	g/L
	GLB	20.0–40.0	g/L
	A/G	1.2–2.4	NA
	ALT	7–40	U/L
	AST	13–35	U/L
	ALP	50–135	U/L
	GGT	7–45	U/L
	AST:ALT	NA	NA
	TBIL	0.0–23.0	μmol/L
	DBIL	0.0–8.0	μmol/L
	IBIL	1.7–15.2	μmol/L
	K	3.50–5.30	mmol/L
	Na	137.0–147.0	mmol/L
	Cl	99.0–110.0	mmol/L
	Ca	2.11–2.52	mmol/L
	P	0.85–1.51	mmol/L
	Mg	0.60–1.10	mmol/L
	GLU	3.89–6.11	mmol/L
	CREA	41.0–81.0	μmol/L
	UREA	3.10–8.80	mmol/L
	URIC	150–350	μmol/L
	Trig	0.56–1.70	mmol/L
	CHOL	2.84–5.69	mmol/L
	HDL-C	1.03–1.55	mmol/L
	LDL-C	1.55–3.36	mmol/L
Laboratory index	APOA1	1.20–1.60	g/L
	APOB	0.80–1.20	g/L
	APOE	2.0–10.0	mg/dL
	APoB/APoA1	NA	NA
	LPa	0.0–0.30	g/L
	LDH	120–250	U/L
	CK	40–200	U/L
	CHE	3930–10800	U/L
	PALB	160–350	mg/L
	TBA	0.0–15.0	μmol/L
	ADA	0.0–20.0	U/L
	GSP	1.15–2.25	mmol/L
	B2MG	0.91–2.20	mg/L
	AFU	14.3–39.9	U/L
	AMY	35–135	U/L
	HCY	0.0–20.0	μmol/L
	LAC	0.50–2.20	mmol/L
	IRON	7.8–32.2	μmol/L
	TIBC	45.0–75.0	μmol/L
	ABO	A/B/AB/O	NA

The range of Symptom duration: ≤ 7 days; 7 days < duration < 30 days;1 month ≤ duration < 3 months; 3 months ≤ duration < 6 months; 6 months ≤ duration < 12 months;1 year ≤ duration; Smoking/Drinking history: includes patients who ever suffered or are suffering; NA, not applicable.

This retrospective study was approved by the ethics review board of the Affiliated Lihuili Hospital, Ningbo University (no. KY2023SL058–01).

### 2.2 Operative technique and rehabilitation after ARCR

To ensure consistency, our organization adopts the same surgical approach and rehabilitation strategy as external verification institutions. The surgery we perform is arthroscopic rotator cuff repair (ARCR) with double-row fixation and arthroscopic acromioplasty. This technique is used for all operations. The procedure consists of the following general steps: (1) After general anesthesia is achieved, left lateral position, affected upper arm abduction traction bracket fixed, Routine surgical area disinfection towel. (2) A small incision is made on the anterior and posterior sides of the affected shoulder. The arthroscope is then inserted through a posterolateral approach and the probe is inserted into the anterior incision to explore the intraarticular tissue. Proliferative synovium is removed and the long head tendon of the biceps brachii is severed. (3) Shape the acromion. (4) The arthroscope is inserted into the subscapular space. A bone bed is created at the greater tubercle of the humerus. Internal rows consisting of one to two 4.5 mm anchors are inserted and external rows consisting of one to two 4.75mm anchors are inserted laterally to fix the ruptured supraspinatus muscle. (5) Close incision after satisfactory arthroscopic probing.

All patients followed the same recovery strategy. Nonsteroidal anti-inflammatory drugs (NSAIDs) were given to relieve inflammation and pain after surgery, patients were advised to avoid traction and maintain an autonomous position. After 1 month later, we will teach the patients to do rehabilitation exercises, including stretching exercises in different directions (front, back, left, right) and wall climbing exercises. NASIDs will be continued if patients experience significant nighttime pain symptoms that affect their sleep. Monthly follow-up appointments were scheduled to monitor patients' posture and ensure proper functional recovery. Generally speaking, the pain symptoms will improve significantly and function will recover significantly after the third month.

### 2.3 Deep learning prediction via EV-GCN

**Figure 2:**
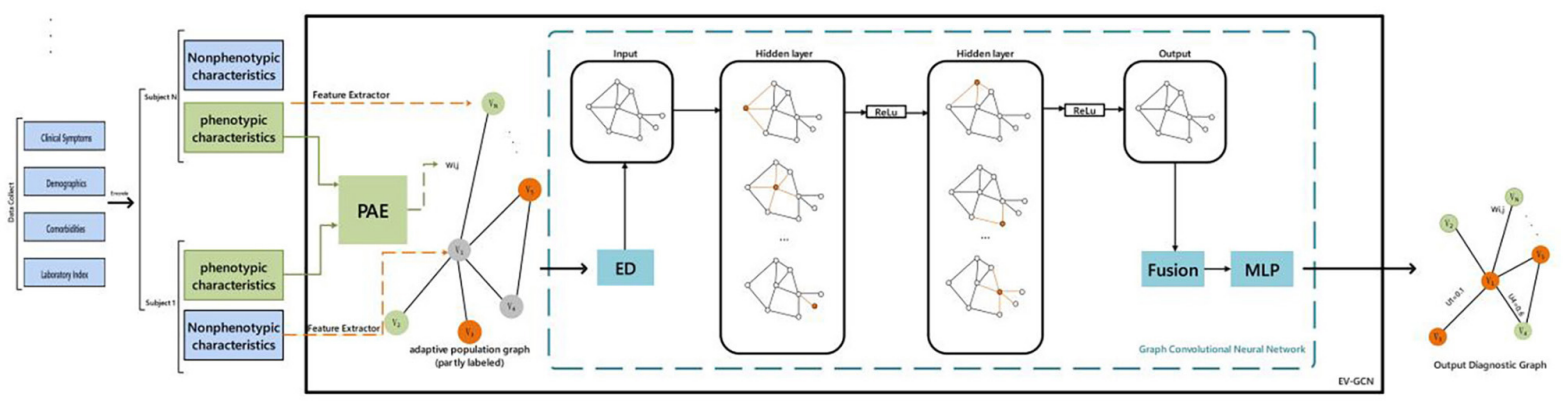
Study overview. PAE, pairwise association encoder; ED, edge dropout; ReLu, linear rectification function. Fusion: vertex-wise concatenation. Colors in the graphs: green and orange - labeled diagnostic values (e.g., healthy or diseased), gray: unlabeled. Ui: predictive uncertainty for subject i.

We implemented the Edge-Variational GCN (EV-GCN) algorithm using PyCharm in python to extract features from clinical data (Huang and Chung, 2022). The overview of the pipeline and the research approach is depicted in Figure 2. This EV- GCN consists of two hidden layers, each followed by ReLU activation to increase non-linearity, and then directed to fusion block and MLP predictor. Phenotypic characteristics are discrete data, that is, gender, clinical symptoms and other dichotomous data. Non-phenotypic characteristics refer to non-discrete data, namely individual laboratory measures. There was a certain difference in the amount of data between the two groups, and we used downsampling to align them.

Construction of adaptive graph: the non-phenotypic features of patients are utilized as data points, and the similarity of phenotypic features of two patients is calculated by the PAE method, which is used as the edge between two data points. V is a finite set of vertices, where |V| = N, E ⊆ V × V is a set of edges, and W is the weight of the edge. The learning function of (xi, xj) is the edge weight wi, j ∈ W between the i-th and j-th vertices. Using a paired associative encoder (PAE) with trainable parameters Ω to model the learning function f: (xi, xj) → R, so that wi, j = f (xi, xj; Ω) (where the parameter Ω is initialized by He Initialization). By using the above idea of constructing edges and points to obtain an adaptive graph (Figure 1), then performing edge dropout, four-layer GC (each hidden layer has a ReLu function), and Fusion and MLP, the result can be output. After applying the conventional edge dropout, convolution, fusion operation, an MLP is constructed: xi, xj are normalized to 0 mean and 1 variance, and the projection network is used to map these normalized values to the latent space hi ∈ RDh (commonly used Dh = 128). The projection network is a multi-layer perceptron (MLP) with one hidden layer (the hidden layer contains batch normalization and dropout operations). The latent feature formula of xi is hi = Ω (2) σ (Ω (1) ~xi + b), where σ is the ReLU function (xi is the normalized input). Finally, the output result is 0 and 1, where 0 means no tearing and 1 means tearing again.

The ratio of training and test sets is 8:2.10-fold nested cross-validation was performed using Stratified K Fold, so there is no explicit validation set. In order to improve convergence and avoid overfitting, hidden layers are combined with batch normalization and dropout. The learning rate was 0.002. Edgedropout was 0.3. Dropout was 0.2. External independent data sets are validated using two-fold validation.

### 2.4 Model evaluation metrics and empirical evidence

We use accuracy, precision, recall, F1 score, loss curve, and confusion matrix for evaluating, discriminating, and calibrating the model performance. The confusion matrix is a two-dimensional model, where the horizontal axis represents predicted labels, which are the classifications obtained by the model's predictions on the data samples, and the vertical axis represents the true labels, which refers to the actual classification of the data collected in this study. By comparing the differences between the true labels and predicted labels, the confusion matrix is able to visualize and display the quantities of various classification outcomes. In addition, the loss function is employed to measure the discrepancy between the predicted outcomes and ground truth. The progressive fitting of the loss function indicates that the model performs well in predicting and comprehending the facts within the dataset. By examining the dataset characteristics (Tables 2, 3), professional medical background, and ethical approval, we guarantee utmost accuracy and soundness of our dataset. The authentic dataset and loss function serve as crucial criteria and foundations for determining the model's adherence to facts.

**Table 2:** Sample characteristics of DL data sets (our hospital).

**Total samples (*n =* 2,372)**	**Eliminated samples (*n =* 741)**	**Total research group (*n =* 286)**	**Eliminated research group (*n =* 78)**	**Total control group (*n =* 2,086)**	**Eliminated control group (*n =* 663)**
**Sample characteristics**	**Partial variables**	**Research group (*****n** =* **208)**		**Control group (*****n** =* **1,423)**	**ALL (*****n** =* **1,631)**
Surgery after Arthroscopic Rotator Cuff Repair	Gender: Male	73 (35.1%)		499 (35.1%)	572
	Gender: Female	135 (64.9%)		926 (65.1%)	1,061
	Age at surgery: ≥60 y	127 (61.1%)		730 (51.3%)	857
	Caused by trauma	66 (31.7%)		297 (20.9%)	363
	Duration of symptoms: ≥6 months	130 (62.5%)		854 (60%)	984

**Table 3:** Sample characteristics of external data.

**Sample characteristics (Form East Medical Center)**	**Partial variables**	**Research group (*n =* 22)**	**Control group (*n =* 248)**	**ALL (*n =* 270)**
Surgery after Arthroscopic Rotator Cuff Repair	Gender: Male	7 (31.8%)	88 (35.4%)	95
	Gender: Female	15 (68.1%)	160 (64.5%)	175
	Age at surgery:≥60 y	5 (22.7%)	102 (41.1%)	107
	Caused by trauma	7 (31.8%)	78 (31.4%)	85
	Duration of symptoms:≥6 months	10 (45.5%)	108 (43.4%)	118

## 3 Results

Overall 2,372 individuals were counted and a total of 147,064 data size were collected, 741 were excluded because of the exclusion criteria and 1,631 were included, of which research group: 208 (35.1% male sex, 61.1% ≥60 years old, 31.7% caused by trauma, 62.5% duration of symptoms≥6 months), control group: 1,423 (35.1% male sex, 51.3% ≥ 60 years old, 20.9% caused by trauma, 60% duration of symptoms≥6 months), 101,122 data size were included at last (Table 2). The experimental group and the control group showed no significant differences in basic variables, such as gender, fraction of elderly people, and fraction of patients with extended symptom duration, in the independent data set.

A total of 270 external independent data sets were collected including 22 cases in the research group (31.8% male sex, 22.7% ≥60 years old, 31.8% caused by trauma, 45.5% duration of symptoms ≥ 6 months) and 248 cases in the control group (35.4% male sex, 41.1% ≥60 years old, 31.4% caused by trauma, 43.4% duration of symptoms ≥ 6 months) (Table 3). The independent data set had a limited number of samples and distinct sample attributes from the model data set, with lower proportions of older people and patients with extended symptom duration in the external data. The 62 preoperative variables included in the two datasets are shown in Table 1, including 4 clinical indicators, 2 demographic indicators, 5 accompanying symptoms, 51 blood biochemical indicators, and their normal ranges and units.

Our DL prediction model achieved 96.93% accuracy,97.18% recall, 99.37% Precision, and 98.26% F1-Score, as verified by ten-fold cross-validation, and the results of the test set (Table 4) showed no apparent anomalies.

**Table 4:** Verification result of EV-GCN deep learning model (our hospital).

	**Accuracy**	**Precision**	**Recall**	**F1-score**
Fold 1	0.9757	0.993	0.9794	0.9862
Fold 2	0.9709	0.9929	0.9862	0.9896
Fold 3	0.9449	0.9929	0.9595	0.9758
Fold 4	0.9714	0.9931	0.9861	0.9896
Fold 5	0.9705	0.9929	0.9861	0.9895
Fold 6	0.9474	0.9929	0.9658	0.9791
Fold 7	0.9752	0.9797	1.000	0.9897
Fold 8	0.9532	0.9929	0.9659	0.9792
Fold 9	0.9706	0.9931	0.9799	0.9864
Fold 10	0.9196	0.993	0.9346	0.9628
Mean	0.9693	0.9937	0.9718	0.9826

Ps: All data are rounded to four decimal places.

**Figure 3:**
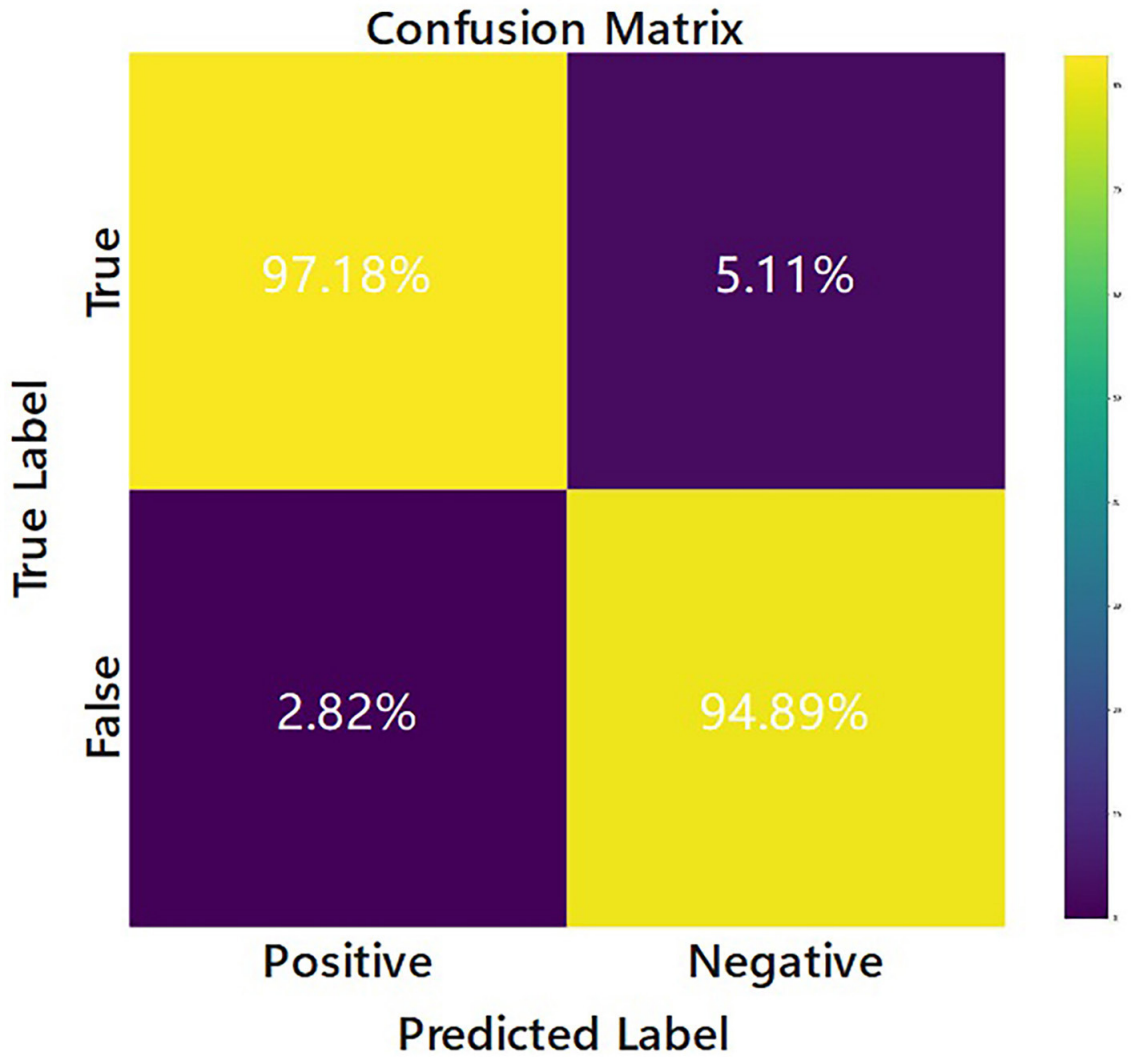
Confusion matrix of the EV-GCN deep learning model classification (Research group of our hospital).

The confusion matrix of the EV-GCN model is shown in Figure 3, where the predicted situation is represented by the horizontal axis and the actual situation is represented by the vertical axis, normalized by column. This visualization shows that 94.89% of the cases of re-tear after arthroscopic rotator cuff repair were correctly predicted as re-tear, and 97.18% of the cases of no difference in follow-up after surgery were correctly predicted as no re-tear.

**Figure 4:**
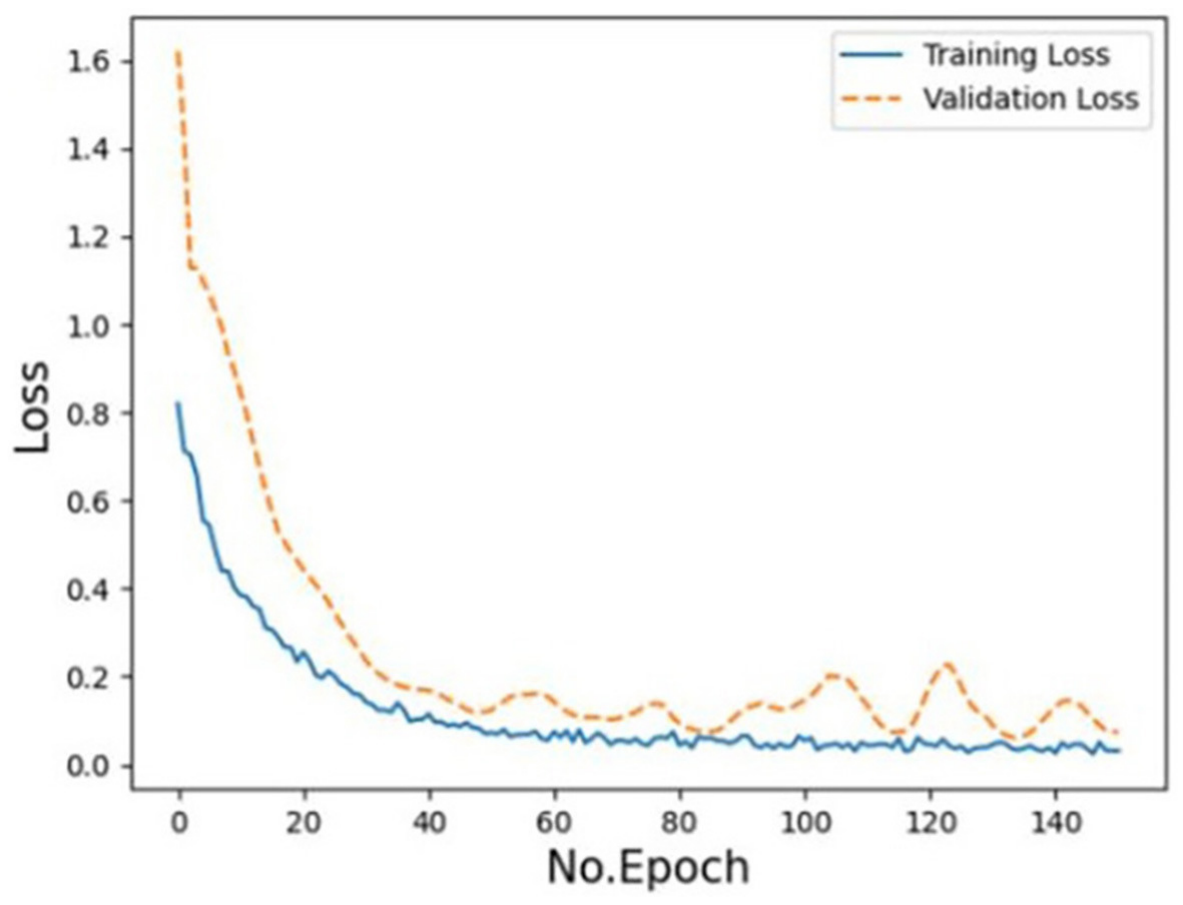
Training and validation loss of the EV-GCN deep learning model (Research group of our hospital).

The training loss and the validation loss of the model displayed a consistent drop and a small overfitting respectively in the loss curve (Figure 4), but they both moved toward convergence progressively. Furthermore, the model was verified by external data. The accuracy rate of prediction is 79.55%, precision is 73.33%, recall is 1, and F1 score is 83.04%.

## 4 Discussion

The main finding of this study is that the graph convolution network (GCN) algorithm can accurately predict rotator cuff tear recurrence after arthroscopic rotator cuff repair (ARCR) before surgery. This idea has broader applications beyond other complications and surgeries, as it also inspires the exploration of combining medical non-image data with graph neural networks. The proposed model achieved 96.93% accuracy, which was validated by independent external data. The findings supported the original hypothesis. Importantly, this study is the first to successfully predict postoperative re-tear of the rotator cuff. The number of arthroscopic rotator cuff repair is still increasing year by year. The conduct and completion of this study will benefit joint surgeons, as well as the understanding and treatment of rotator cuff injuries.

The formulation of inclusion and exclusion criteria in the experiment is based on clinical practice. As 90% of rotator cuff injuries involve the supraspinatus muscle, the experiment decided to focus on this muscle to enhance accuracy. Typically, within 3 months after ARCR, the tendon has not fully reconstructed. Tears occurring after 12 months are classified as new rotator cuff injuries rather than postoperative re-tears. Anchor fixation alone is often inadequate for repairing large, full-thickness rotator cuff tears. The use of tendon graft or other techniques differs significantly from anchor fixation. Small, superficial rotator cuff injuries do not necessitate anchor fixation and cannot be compared to conventional rotator cuff repair methods, thus leading to their exclusion.

To assess the performance of the model, we generated the loss curve of the model, as shown in Figure 4. The curve gradually converged, and the model generalized well with good reliability. This indicates that the model could learn the intrinsic characteristics of the data, but the curve was not fully fitted. We predicted that increasing the amount of data would make the model more accurate. According to our analysis, this might be because (1) GCN node classification would deteriorate due to different data distributions; (2) node information was aggregated but not transmitted back, and too much unlabeled information would affect the performance of the whole network; (3) the amount of external data was small and unbalanced. We calculated the sensitivity and specificity values and then constructed the confusion matrix to analyze the performance of the model, as shown in Figure 3. In general, a performance above 90% is considered “excellent” (Hosmer et al., 2013). The results depicted in this figure demonstrate the model's strong performance in this classification task, characterized by high discriminability, accuracy, quality, robustness and generalizability.

At the beginning of the study, we conducted *t*-tests and homogeneity of variance tests on the data. The analysis revealed that only a few characteristics exhibited *p* < 0.05, indicating no statistically significant difference between the two groups. We attempted commonly used artificial intelligence algorithms such as Bayesian and Random Forest, but the results were unsatisfactory (see Supplementary material). The complex and uncertain relationship between nodes and the diversity of medical data are more suitable for artificial neural networks and other deep learning algorithms to explore the internal relationships, especially the Edge-variational GCN used in this study. This study confirms the great role of EV-GCN construct in clinical computer-aided diagnosis. Subsequent researchers can try to add Monte-Carlo edge dropout to the model to estimate the predictive uncertainty related to the graph topology.

The primary objective of our study was to predict the risk of re-tear prior to surgery. To achieve this goal, we specifically selected variables that were both preoperative and comprehensive in nature. Although previous studies have identified operative duration, biceps procedure, postoperative UCLA score, number and size of RCTs as risk factors for re-tear (Le et al., 2014; Diebold et al., 2017; Lee et al., 2017), we did not collect these data. BMI was a significant (*P* < 0.001) risk factor for re-tear after rotator cuff surgery (Ateschrang et al., 2018), but the BMI data in our hospital system were incomplete, so we excluded it. Preoperative imaging also had important reference value (Saccomanno et al., 2015), but we could not match the data and images for the large number of cases. Other factors that have been reported to affect re-tear after ARCR include hyperlipidemia, smoking history, age, gender, preoperative symptoms, and affected limb (Robinson et al., 2013; Namdari et al., 2014; Garcia et al., 2017; Park et al., 2018; França et al., 2021; Kim et al., 2021), but few studies have systematically examined routine laboratory tests before surgery. We hypothesized that these data could reflect the patient's physical condition over time and influence rotator cuff healing. Therefore, in order to maximize the acquisition of features, we collected as many as possible 62 preoperative variables, including clinical and basic laboratory indicators. Most of these indicators were based on standard medical tests (Table 1), offering high objectivity and consistency.

Graph convolutional network (GCN) applies convolution operations to graphs, utilizing the topological structure and node features of graphs for information propagation and feature extraction. Medical data often exhibit complex interrelationships that are challenging to process directly using simple methods. The dataset composed of 62 preoperative variables of this model is a typical example of non-Euclidean data. The EV-GCN algorithm contains a unique edge-dropout method, which is similar to dropout. When the model has too many parameters and not enough training samples, the trained model is prone to overfitting. This method will make the model more generalizable, because it will not rely too much on some local features. It is very helpful for the construction of medical models with complex relationships and difficult data collection.

Artificial intelligence technology in the field of rotator cuff tears is often limited to image recognition, classification, and diagnosis, with less research focused on postoperative complications. Recently, Shinohara et al. (2023) attempted to use machine learning (ML) algorithms to predict postoperative re-tears of the rotator cuff. Among the five algorithms applied, the highest prediction rate achieved was only 87%, based on statistical parameters from 353 collected cases. Cho and Kim (2023) innovatively used intraoperative arthroscopic images to predict postoperative re-tears, but the study included only 580 patients and applied three traditional deep learning (DL) algorithms, with the highest prediction rate reaching 91%. In contrast, our study benefits from a large sample size, comprehensive variable range coverage, systematic and internationally standardized variable indices, innovative algorithms, and high prediction rates. It has also undergone external validation, providing valuable assistance in formulating clinical decisions.

This study has several limitations: (1) MRI images of rotator cuff injury were not included. Because we were not sure whether the prediction model could be successfully established. (2) This was a retrospective single-center study. However, the results of this study are credible, with a large amount of data and verified by external data. (3) The number of re-tear cases was small compared with normal cases. This is because the symptoms of most patients can be significantly relieved after surgery, and the situation of re-tear is less. (4) Deep learning techniques, including graph convolutional networks, are generally uninterpretable due to the black-box phenomenon, while medical treatment emphasizes interpretability. (5) Due to technical reasons, we were unable to construct the ROC curve, which makes the experimental results less coherent. We demonstrated the correlation between the overall health status of the body and the postoperative re-tear of the rotator cuff using 62 preoperative variables, which are non-Euclidean data. However, the specific clinical relevance still requires further study. These are exactly what we are going to do next. MRI images can describe the characteristics of cases from another modality. More data, a wider range of sources, and more balanced data sets are helpful to improve the accuracy of the model. Interpretable and clinically relevant conclusions can also facilitate the application of deep learning techniques in medical practice.

The application of deep learning technology in medicine encounters various challenges, such as (1) Low-quality data (2) Lack of explain ability due to the black box phenomenon (3) Inadequate integration with existing EHR systems. We addressed some of these challenges in our research design, but others remain unresolved. In our future work, we will focus on constructing multimodal models that integrate data and images. However, we must also acknowledge the importance of interpretability. In the healthcare field, which values logic and rigor, the “black box effect” of artificial intelligence severely limits its development in the medical domain. This is presently an active challenge in applied research. In the future, we will investigate potential methods for improving interpretability: (1) Introducing specific network layer structures or mechanisms to ensure clear interpretations of each hidden layer's outputs on the model's final predictions. (2) Visualizing the prediction layer and utilizing attention mechanisms to visualize regions of interest. (3) Exploring the design of interpretable attention mechanisms that incorporate expert knowledge, forcing the model to focus on specific lesion areas or disease features, and enabling the attention mechanism to be explained based on medical knowledge. Achieving the translation of fundamental research will require collaboration between clinical physicians and computer scientists.

In conclusion, a deep learning GCN algorithm was used to develop a prediction model for re-tear after ARCR, which was simple and practical with excellent performance. This model enables clinicians to forecast postoperative results and make informed decisions regarding the adoption of more reliable fixation methods during surgery, as well as the implementation of a more stable rehabilitation process in the early postoperative period. The model has proven effective and holds practical value. By successfully predicting re-tear after rotator cuff repair, this idea deserves promotion to broaden its application scope.

## Data availability statement

The raw data supporting the conclusions of this article will be made available by the authors, without undue reservation.

## Ethics statement

The studies involving humans were approved by the Ethics Review Board of the Affiliated Lihuili Hospital, Ningbo University. The studies were conducted in accordance with the local legislation and institutional requirements. The human samples used in this study were acquired from a by- product of routine care or industry. Written informed consent for participation was not required from the participants or the participants' legal guardians/next of kin in accordance with the national legislation and institutional requirements.

## Author contributions

ZheZ: Conceptualization, Investigation, Methodology, Validation, Writing—original draft. CK: Writing—original draft. ZhiZ: Software, Validation, Writing—original draft. YC: Writing—original draft. HW: Writing—original draft. JD: Writing—original draft. MH: Writing—original draft. BL: Writing—original draft. MZ: Writing—original draft. JL: Writing—original draft. SD: Writing—original draft. YD: Resources, Writing—review & editing. ZP: Resources, Writing—review & editing.
